# Improving urban emergency medical service systems through brownfield transformation in Huangshi, China

**DOI:** 10.1038/s41598-024-66080-3

**Published:** 2024-06-28

**Authors:** Zhiping Liu, Yingxue Feng, Jing Li, Haoyu Tao, Zhen Liu, Xiaodan Li

**Affiliations:** 1 State Key Laboratory for Tunnel Engineering, China University of Mining and Technology (Beijing), D11, Xueyuan Road, Haidian District, Beijing, China; 2 School of Mechanics and Civil Engineering, China University of Mining and Technology (Beijing), Beijing, 100083 China

**Keywords:** Urban brownfields, EMS, Service radii, Suitability of reconstruction, Transport time, Huangshi, Sustainability, Epidemiology

## Abstract

A comprehensive emergency medical service (EMS) system significantly enhances a city’s capacity to prevent and mitigate disasters. Using Huangshi as a case study, this research evaluated the service radium coverage rate of the current EMS system by examining its transport capacity, population density, and prevalence rate, finding it to be only 61.49% with an inefficient spatial layout. To address this, we proposed transforming urban brownfields into EMS parks. By selecting the most suitable brownfields based on capacity and service radius, we increased the coverage rate to 90.21%. We introduced a new "consultation-referral" model, where existing EMS facilities serve as pre-diagnosis and triage centers, and the urban brownfield EMS parks function as isolation and centralized treatment centers. GIS network analysis confirmed the feasibility, showing all transit times to be under 30 min. The methodology outlined in this study—comprising "demand assessment, supply optimization, and feasibility verification"—not only strengthens the city’s EMS system but also facilitates the renewal of urban brownfields. This approach can serve as a valuable reference for enhancing EMS systems in other cities.

## Introduction

Since the beginning of the twenty-first century, there have been eight major public health emergencies of international concern. These include the Severe Acute Respiratory Syndrome (SARS) in 2003, the H1N1 influenza pandemic in 2009, the polio epidemic in 2014, the Ebola epidemic in West Africa in 2014, the Zika virus epidemic in 2015–2016, the Ebola epidemic in Congo in 2018 and 2019, the COVID-19 outbreak in 2020 and the monkey pox outbreak in 2022^1^. The outbreak of COVID-19 in 2020 was one of the a major public health crises. As of February 28, 2023, COVID-19 infections have been reported in 222 countries worldwide, with a total of 6.644million confirmed cases^2,3^. The COVID-19 outbreak has seriously affected the social and economic operations of countries around the world and posed great challenges to the cities’ EMS systems^4^. An EMS system, which consists of EMS facilities, is defined as a “Formalized pre-hospital care, provided by emergency care professionals who respond to medical emergencies within a well-defined jurisdiction”^5^. This EMS system plays a critical role in emergency systems by providing timely and safe on-scene evaluation, stabilization, and transport of patients to an appropriate facility^6,7^. When people experience a significant public health emergency, open spaces, such as parks and other open areas, could serve as refuge places that satisfy survival needs^8^. In this context, most developed countries have implemented the concept of urban disaster prevention parks to improve the EMS system^9–12^. Japan has started to plan and develop urban disaster prevention parks due to the frequency of disasters such as fires and earthquakes^13–15^. The City Park Law in Japan (1993) was the first government-formulated policy that gave city parks the status of EMS systems^16^, and defined the following park functions: (1) during ordinary times, provide recreation, sightseeing, and cultural entertainment; (2) in case of disasters, provide shelters and emergency medical service, and can mitigate or prevent the spread of disasters and potential secondary disasters^17^. After the Great Hanshin Earthquake, the Japanese Ministry of Construction put forward two disaster prevention requirements for the construction of urban parks in 1995. First, increase the number of disaster prevention parks in the old part of the city to form a network system^18^. Second, general parks should be transformed into disaster prevention parks to improve their disaster prevention capabilities^19,20^.

Numerous studies have shown that during frequent urban disasters such as earthquakes, floods, and major public health events, the supply–demand relationship between the potential refugee population and its need for refuge space is crucial in EMS park planning^21,22^. The core issue for both EMS facilities and EMS parks is the residents’ arrival time; if too long, the risk increases. Therefore, each EMS facility and EMS park must have an appropriate service radius to provide effective services^23,24^. Evaluating the service radii is critical for ensuring residents’ safety and implementing a comprehensive EMS system^25^. Many researchers have studied the location and layout of EMS parks, focusing on their effectiveness, safety, and accessibility^26,27^. They have refined the evaluation indicator system to include metrics such as effective shelter area, service area, open space ratio, proximity to hospitals, fire stations, large supermarkets, and distance from hazards and earthquake rupture zones^28–30^. Although these indicators are designed to assess the suitability of EMS parks, most of the existing evaluation factors focus on area and distance, ignoring road conditions during transport. This indicator determines the degree of danger faced by residents. Studies have shown that as the transport time increases, the threat to residents increases^31–33^. Therefore, in this study, the transport time was selected as a validation criterion of the EMS park site. Remote sensing (RS), Global Positioning System (GPS) and Geographic Information Systems (GIS), collectively called “3S system”, are important tools in this field^34^. Li et al. (2020)^35^ proposed the nearest neighbor method in combination with GIS, considered road networks and census data, and evaluated the service extent of refugee parks in Changchun. Shi et al. (2023)^36^ used GIS network analysis technology to evaluate the accessibility of emergency medical aid for kindergartens and nursing homes in the Erqi District of Zhengzhou under different pluvial flooding scenarios. Yin et al. (2019)^37^ presented a scenario-based study that assessed the vulnerability of emergency services in case of school-related emergencies in the city center of Shanghai, China, by means of GIS-based accessibility mapping. Past research in disaster prevention and control has shown that rational and effective planning of EMS sites is an effective measure to improve hazard prevention during catastrophic events^38,39^. In combination with real-time ground and statistical data, the 3S system can predict the evacuation time and simulate the conditions for the affected residents and facilities during disasters and can provide visual aids for decision-making^40–42^.

**Figure 1:**
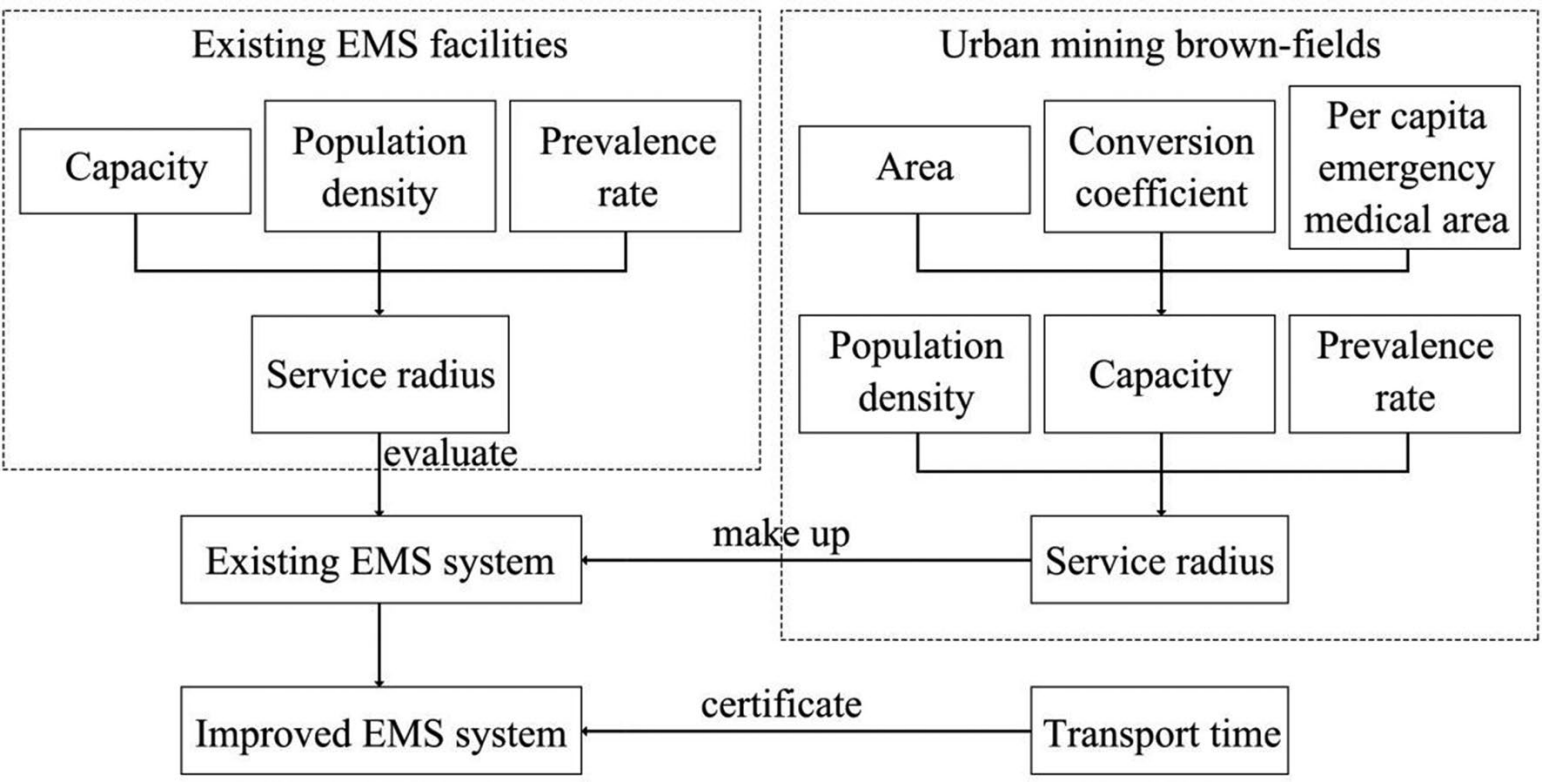
Research flowchart.

Considering the diverse needs of the evacuees and the special characteristics of vulnerable groups, this study selected Huangshi for the case study, established an evaluation index system with four criteria: capacity, population density, prevalence rate, and transport time. This study developed a comprehensive evaluation model of existing EMS facilities and urban brownfield EMS parks. It also analyzed the interaction between the distribution and the service functions of EMS sites and provides recommendations for EMS sites optimization, as shown in Fig. 1. The main contributions are as follows:

1. From the perspective of population density, prevalence rate and capacity of EMS facilities, an evaluation model has been set up to determine the service radii provided by existing EMS facilities.
2. An optimized EMS system is proposed, which transforms the urban brownfields into EMS parks. After applying the adequate conversion coefficients for area, per capita EMS area, and the service radii, the results from the study demonstrate that the new EMS system would provide the larger capacity and service radii than the currently existing EMS facilities.
3. Based on the traditional location selection method, the feasibility of the transportation mode between existing EMS facilities and urban brownfield EMS parks is verified by using the GIS network analysis from the perspective of transport time. On this basis, this study proposes a “consultation—referral” model and discusses the enhancement effect on the existing EMS system.

## Materials and methods

### Study area

The city if Huangshi is located in the southeast of Hubei Province, on the south bank of the Yangtze River in China, with a total area of 4583 km^2^ and jurisdiction over 6 districts (Xialu, Xisaishan, Huangshigang, Tieshan, Daye and Yangxin). The population distribution among these districts varies greatly; the population density is highest in the northeastern part of the city; it occupies 4.96% of the area and accommodates 28.19% of the population, with Huangshigang having the highest population density with 8053.33 people/km^2^. Huangshi is an old industrial city in China with a history of more than three thousand years of mining and smelting culture. However, in the past decade, the mineral resources of Huangshi have been gradually depleted, and signs of urban post-industrialization have progressively emerged. At the same time, brownfields in the city started to emerge in large numbers. These idle brownfields are the cause of industrial pollution, resource exploitation, or improper disposal of waste. Alternative industries have yet to form, and urban industrial transformation is difficult, which is significant hindering the sustainable development of Huangshi. During the COVID-19 outbreak, Huangshi has exposed the problems caused by a malfunction urban EMS system, such as a shortage of emergency public medical resources and the severe shortage of local material supplies, which puts the life and health of urban residents at risk, making the construction of EMS parks in Huangshi particularly important.

Based on the above criteria, this study assessed the transformation potential of the Huangshi brownfields into EMS parks with the objective to improve the urban EMS system.

### Data source

The data sets used in this study mainly consist of two parts.

#### Part 1: Basic data

**Figure 2:**
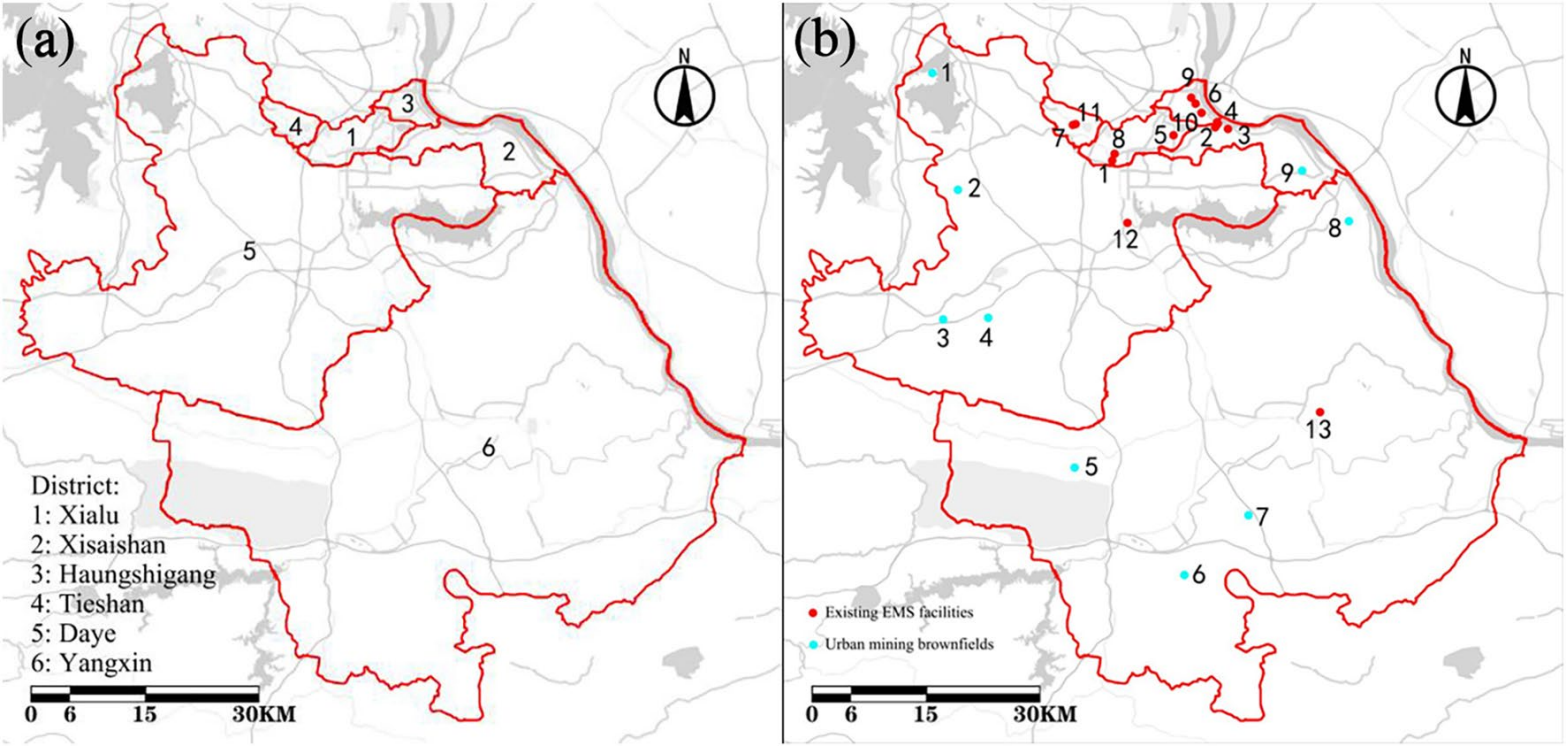
The basic information of Huangshi. (**a**) Map of each district of Huangshi; (**b**) Distribution of existing EMS facilities and urban mining brownfields in Huangshi.

This part part of data mainly provides geographic information data and disaster statistics of Huangshi (Table S1, Fig. 2a). The geographic information data, including the name, distribution, area, population and population density of the six districts in Huangshi, were mainly retrieved from the National Geographic Information Public Service Platform and the Huangshi Statistical Yearbook (2021), published by the National Bureau of Statistics (http://www.huangshi.gov.cn/sjfb/tjnj). The disaster statistics of the cumulative number of confirmed cases, as reflected in this study, mainly come from the official website of the Huangshi Municipal Health Committee (http://wjw.huangshi.gov.cn, as available on 9 March, 2020, the first peak in COVID-19 cases).

#### Part 2: The data of existing EMS facilities and urban brownfields

The data on the existing EMS facilities, including the 13 designated EMS facilities set up in Huangshi to treat patients during the COVID-19 outbreak, were related from the Official Website of Huangshi Radio and Television (http://www.hsgd.net.cn.) (Table S2). The data also contain EMS capacity for temporary observation, mild and severe cases. The data on urban brownfields, including coordinates and area (Table S3), were mainly gathered by field research using handheld GPS system. Distribution of existing EMS facilities and urban brownfields in Huangsh (Fig. 2b).

### Methods

This study was conducted in 3 steps as follows.

#### Step 1: Measuring the service radii coverage rate of existing EMS system

1. Measuring the service radii of existing EMS facilities

For each existing EMS districts *i*, we determined the capacity (*P*) of the EMS district (*i*), the population density (*d*), and the prevalence rate (*m*) around the EMS district *i*. We computed the service radiu (*L*) of the EMS district *i* by Eq. (1):

1 $$ Li = \sqrt {\frac{Pi}{{\pi \times di \times mi}}} $$

where the prevalence rate (*m*) is an important index which determines the service radii of EMS facilities. It ensures the accuracy of the service radii only if it is reasonably predicted. Yan et al. (2011)^43^ estimated that, in 2020, the number of suspected cases that required isolation and observation was about 1.5 times the number of confirmed cases. Patients under temporary observation and confirmed cases need to be isolated during major public health emergencies. In this study, the case numbers taken into account are the total number of people who need to be separated. This translates into an effective total number of people in the region that is 2.5 times the number of diagnosed cases. The prevalence rate in the region equals the number of confirmed patients × 2.5/over the respective regional area.

2. Calculating the service radii coverage rate of existing EMS system

To enhance a city's EMS system, referencing the research of Yang et al. (2020)^44^ and Grot et al. (2022)^45^, the service radius coverage rate of EMS facilities is a key evaluation standard. We calculated the service radius coverage rate of emergency EMS facilities (*ID*_*COV*_) using Eq. (2):

2 $$ IDCOV = \sum\limits_{k = 1}^{m} {SCOV/S} $$

where *S*_*cov*_ is the coverage area of the service radius of a certain type (or level) refuge in an evaluation unit, and *S* is the evaluation unit's total area.

#### Step 2: Determination of the suitability of urban brownfield EMS parks

This approach was used to compute the service radii (*L*) of each urban brownfield EMS parks *i* through two processes:

1. Determination of the capacity of urban brownfield EMS parks

For each urban brownfield park *i*, we determined the area (*S*), the conversion coefficient (*e*) and the per capita EMS area (*r*) of the urban brownfield *i*, and computed the capacity (*P*) of the urban brownfield park *i* by Eq. (3):

3 $$ Pi = \frac{Si \times ei}{{ri}} $$

where *e* is the conversion coefficient of the urban brownfield EMS parks. In addition to designating brownfields as EMS parks sites for emergencies, the daily use of the urban brownfield parks should also be considered, such as the establishment of botanical gardens, exhibition halls, amusement parks, parks, etc. The brownfields could be equipped with simple and movable facilities, facilitating their transformation into EMS parks. For this study, we estimated that the open space area of EMS sites accounts for 10% of the total urban urban brownfield parks area.

*r*, the per capita emergency medical area of the urban brownfield parks, is a crucial index related to normal and orderly operation of the emergency medical site. This study takes the per capita EMS area of 49.86 m^2^/person for the region, based on the design data of the Wuhan “Huoshenshan” hospital and the “Leishenshan” hospital.

2. Determination of the service radii of the urban brownfield parks

For each urban brownfield EMS park *i*, we estimated the capacity (*P*) of the urban brownfield park *i*, the population density (*d*), and the prevalence rate (*m*) around the urban brownfield park *i*, and calculated the service radii (*L*) of the urban brownfield EMS sites *i* also using Eq. (1).

#### Step 3: Determination of the travel time between urban brownfield EMS parks and existing EMS facilities

Access to timely surgical care is of particular importance because delays may lead to increased patient health risks or life-threatening emergencies with increased complications, mortality, and costs^46^. This study adopted the index of transport time to prove that the location of these urban brownfield EMS parks can improve Huangshi’s EMS system.

Planners need evidence to make informed ‘location allocation’ decisions; this process has traditionally been supported by GIS analyses of accessibility to facilities^47^. The geographic information system (GIS) can be used to manage and analyze spatial data to address this problem. GIS provides a variety of tools for location-based services and optimization of site selection^48^. Studies by Pirnazar and Moslem et al.^49,50^ have proved that GIS is a robust tool for mapping and visualizing location characteristics and selecting adequate location-based services. The spatial analysis feature of GIS provides a tool for selecting the appropriate healthcare site.

The network analysis tool in GIS can be used to calculate the distances and travel times between locations or points via linear networks, like roads. Sets of specific locations are often described as “supply” or “demand”. In this study, the urban brownfield parks were defined as “demand points”, and the existing EMS facilities were the “supply points”. The network distance between the 6 urban brownfield parks and 13 existing EMS facilities was determined by the Network analyst, resulting in a time matrix from each existing EMS facility to each urban brownfield EMS park was generated.

### Ethical approval

All methods in this article were carried out in accordance with relevant guidelines and regulations. This article does not involve animal and/or human testing, and it does not also involve human participants in this research. Then, this work does not apply for statement to confirm that all experimental protocols were approved by a named institutional and/or licensing committee.

### Consent to participate

Informed consent was obtained from all individual participants included in the study.

## Results and discussion

### The evaluation of existing EMS facilities

For this study, the capacity of existing EMS facilities, the population density and the prevalence rate around the site were selected to jointly determine the service radii of these facilities (Eq. 1), as detailed in Table S4.

**Figure 3:**
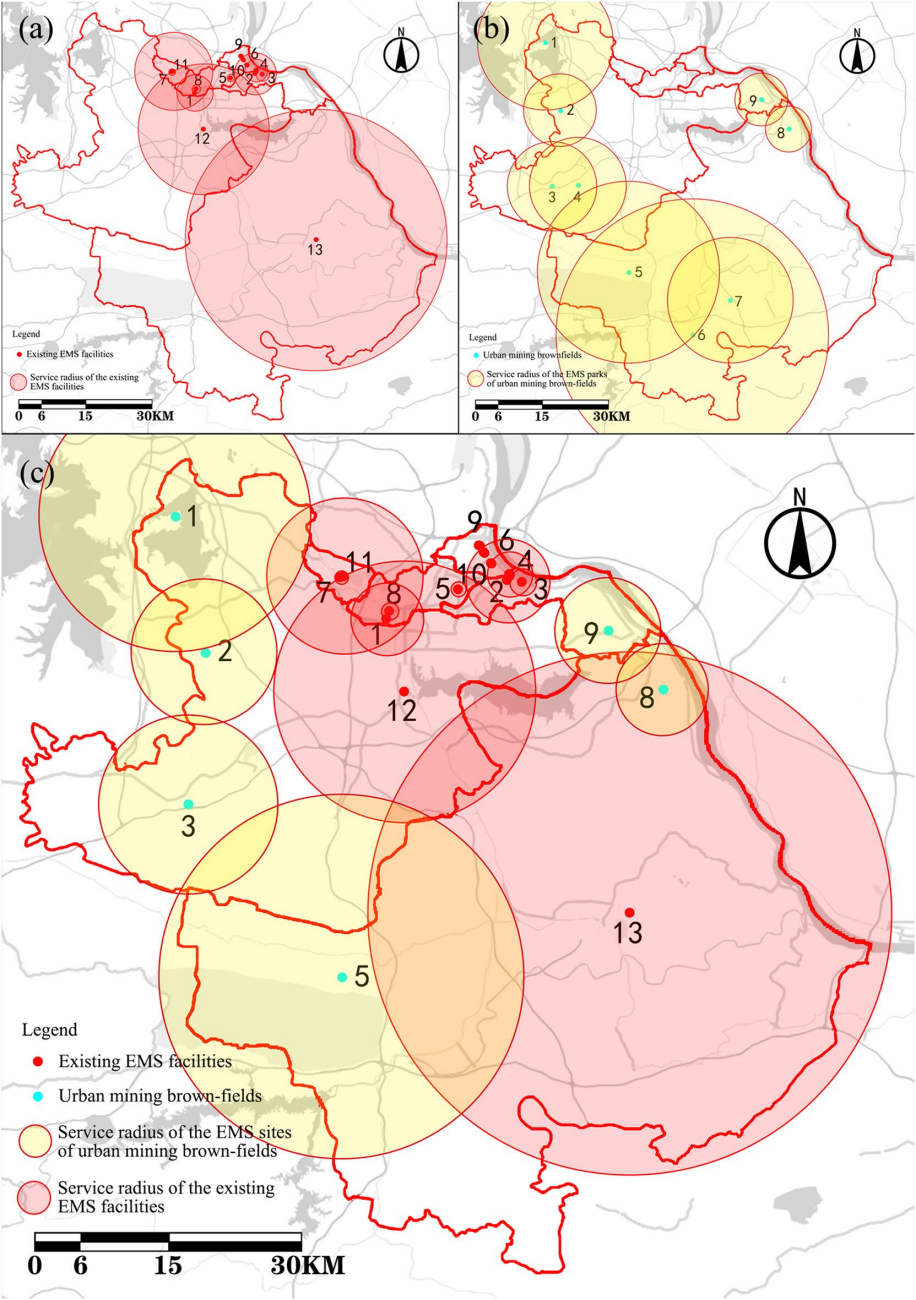
Service radii of existing EMS facilities (**a**), urban mining brownfield EMS parks (**b**), and the improved EMS system (**c**).

Figure 3a shows, during the COVID-19 outbreak, 13 designated EMS facilities were set up in Huangshi to treat COVID-19 patients. In the 13 designated EMS facilities, a total of 1775 isolation ward beds were set up, including 914 beds in the northern areas, 511 beds in the center, and 350 beds in the southeast. These EMS facilities in Huangshi are inadequate and not evenly spaced. They are mainly concentrated in Xialu and Huangshigang; few of them are in Tieshan and Xisaishan, and Daye and Yangxin only have one. In contrast, in the northern area, the distribution is relatively dense, and many EMS facilities have overlapping service. There is only one EMS facility in the southeastern region; there are none in the northeast and west.

The service radius covers only 61.43% of the city of Huangshi, which is both insufficient and unevenly distributed. In improving the coverage and building a fully functional EMS system, it is critical to add EMS parks in the southeast, the northeast, and the west of Huangshi.

### The suitability for the transformation of urban brownfields

For this study, the area of urban brownfield, the conversion coefficient, the per capita emergency medical area, the population density, and the prevalence rate around an urban brownfield were selected to assess the suitability of transforming this particular urban brownfield into an EMS park. The service radii of each urban brownfield park could be calculated by Eqs. 2 and 3, as detailed in Table S5.

Figure 3b illustrates the problem of too many overlapping service radii and too concentrated urban brownfield parks in some areas, brownfield resulting in a waste of resources. Therefore, three of the urban urban brownfields numbered 4, 6 and 7, were removed, and the remaining six urban brownfields were retained. Together with the existing EMS facilities, they form the EMSS in Huangshi, which raises the service radius coverage rate in Huangshi to 90.21% (Fig. 3c).

### Transport analysis between urban brownfield EMS parks and existing EMS facilities

#### Transport time analysis

**Figure 4:**
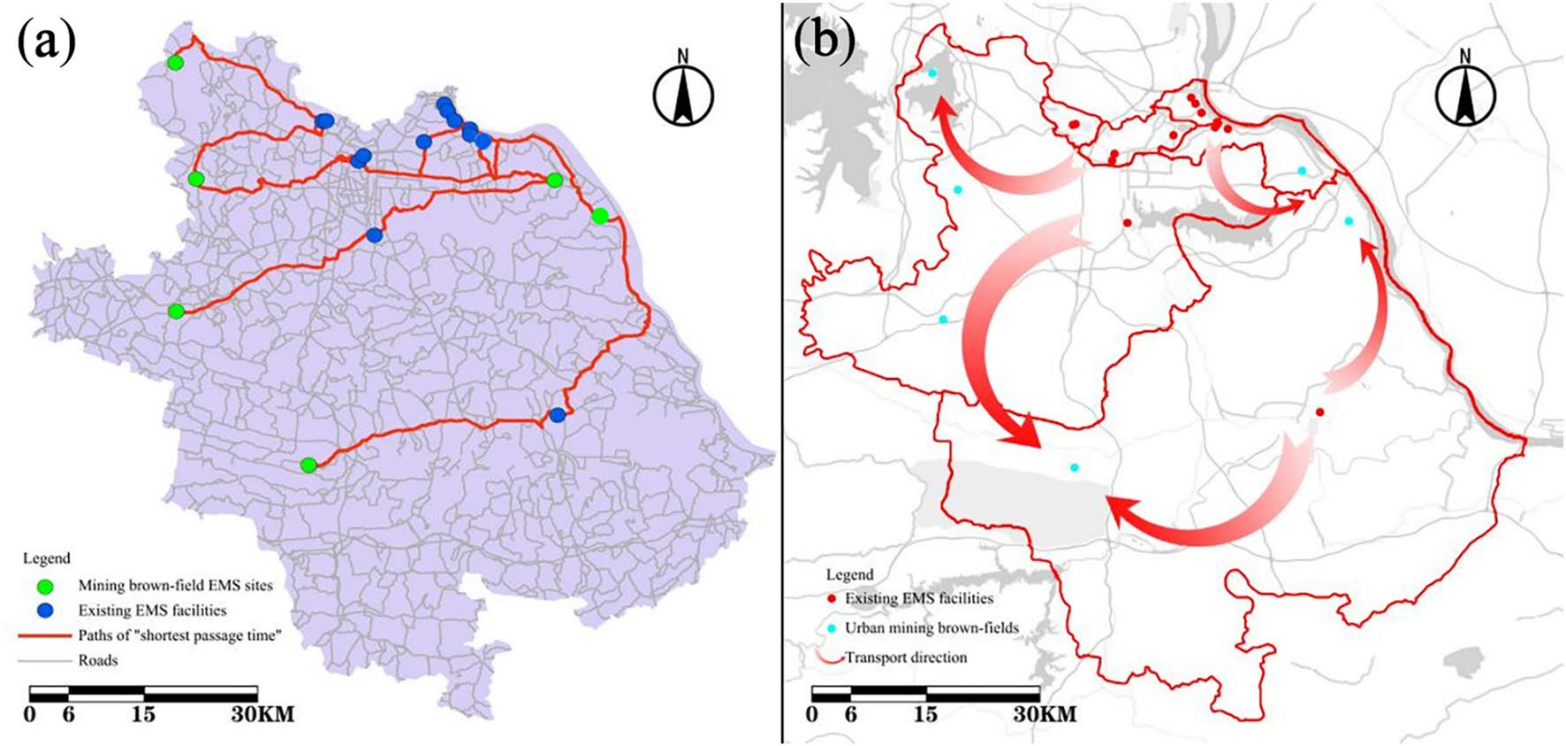
The transport path (**a**) and direction (**b**) of the model of “consultation-referral”.

The trend of increasing ambulance transfer time has been further exacerbated by infectious disease outbreaks such as influenza and the COVID-19 pandemic^51,52^. This assertion is supported by previous research that found that extended ambulance transfer time has a negative impact on both the EMS system and the patients^53^. The term “ambulance offload delay” (AOD), refers to the extended time starting from ambulance arrival at the hospital to the time that patient care is transferred to the emergency department staff^31,32,54^, It was shown that delays longer than 30 min lead to worsening patient outcomes and higher hospital admission rates^55^. Which is in agreement with reports by Leira et al. (2012)^56^. By setting the condition of "Minimum Time Cost (MTC)", specifying that 1 “Supply point” corresponds to 2 “Demand point” and using network analysis, the paths of "shortest passage time" between urban brownfield EMS parks and existing EMS facilities could be obtained (Fig. 4a).

According to the Table 1, from the perspective of transport time, the time is mainly lies within the range of 15–22 min, accounting for 76.92%; the longest travel time is 29.3 min, less than the 30-min limit. From the perspective of transport destinations, the transfer from the existing EMS facilities are mainly concentrated in urban brownfield EMS parks 8 & 9, which are also the two largest urban brownfield EMS parks with capacities of 301 and 401 persons respectively. This demonstrates that it is feasible to develop a transport model from existing EMS facilities to urban brownfield EMS parks for people with major health emergencies.

**Table 1:** The travel time of each path.

No.	Supply points	Demand points	Transport time (min)
1	Existing EMS facility 1	Mining brown-field EMS park 2	19.5
2	Existing EMS facility 1	Mining brown-field EMS park 9	19.3
3	Existing EMS facility 2	Mining brown-field EMS park 8	17.1
4	Existing EMS facility 2	Mining brown-field EMS park 9	12.5
5	Existing EMS facility 3	Mining brown-field EMS park 8	17.1
6	Existing EMS facility 3	Mining brown-field EMS park 9	11.1
7	Existing EMS facility 4	Mining brown-field EMS park 8	17.0
8	Existing EMS facility 4	Mining brown-field EMS park 9	12.4
9	Existing EMS facility 5	Mining brown-field EMS park 8	21.0
10	Existing EMS facility 5	Mining brown-field EMS park 9	16.4
11	Existing EMS facility 6	Mining brown-field EMS park 8	19.6
12	Existing EMS facility 6	Mining brown-field EMS park 9	15.0
13	Existing EMS facility 7	Mining brown-field EMS park 1	20.6
14	Existing EMS facility 7	Mining brown-field EMS park 2	16.9
15	Existing EMS facility 8	Mining brown-field EMS park 2	19.6
16	Existing EMS facility 8	Mining brown-field EMS park 9	19.6
17	Existing EMS facility 9	Mining brown-field EMS park 8	20.5
18	Existing EMS facility 9	Mining brown-field EMS park 9	15.9
19	Existing EMS facility 10	Mining brown-field EMS park 8	18.9
20	Existing EMS facility 10	Mining brown-field EMS park 9	14.3
21	Existing EMS facility 11	Mining brown-field EMS park 1	20.9
22	Existing EMS facility 11	Mining brown-field EMS park 2	17.2
23	Existing EMS facility 12	Mining brown-field EMS park 3	21.2
24	Existing EMS facility 12	Mining brown-field EMS park 9	20.6
25	Existing EMS facility 13	Mining brown-field EMS park 5	27.0
26	Existing EMS facility 13	Mining brown-field EMS park 8	29.3

#### Analysis of the model of “consultation and referral”

Currently, the existing EMS facilities in Huangshi are located in the northeastern part of the city, which is the central urban area of Huangshi. There are numbers surrounding residential and commercial areas; and the population is large and dense, prone to cross infection and not conducive to a centralized infectious disease isolation and diagnostics center. The use of these urban brownfields to establish centralized isolation, diagnostics and treatment centers has natural spatial advantages: these urban brownfields EMS parks have less communication with cities and are relatively independent and stand-alone due to their remote location. These sites can guarantee the normal operation of the city to a greater extent and reduce the possibility of urban residents being cross-infected, which meets the special needs for isolation and diagnosis during major public health emergencies. At the same time, the urban brownfield EMS parks have a large area, which meets the basic land needs for establishing isolation, diagnosis, and treatment areas.

The study presented in this study has the objective of establishing a comprehensive and systematic EMS system for Huangshi, providing the city with the tools required for quick and effective response during major public health emergencies (Fig. 4b). This approach requires the physical combination of the existing EMS facilities and the urban brownfield EMS parks. The existing EMS facilities are used as pre-diagnostics and triage centers, taking advantage of their location in the main urban area, dense surrounding population and convenient transportation. Patients can be diagnosed nearby, which reduces potential risks of cross-infection among patients during transport. The urban brownfield EMS parks are mainly used as centralized isolation and diagnostics centers, with centralized and unified patient management. Their natural geographical advantage at the city’s outskirts is used to minimize the impact on everyday productivity and life of the city and to ensure the life, health, and safety of urban residents.

Based on the transport time in Table 1, the capacity of existing EMS facilities in Table S2, and the capacity of urban brownfield EMS parks in Table S5, the following transport rules were established:

- Existing EMS facilities 3, 5, 6, 7, 8, 9, and 10 have capacities of 24, 10, 9, 11, 12, 9, and 9, respectively. Due to limited resources, these facilities will serve only as pre-diagnosis and triage centers, without isolation wards. Patients from facilities 3, 5, 6, 8, 9, and 10 will be transferred to urban brownfield EMS park 9, which has the largest capacity (401). Facility 7, located in the city’ s northwest corner, will transfer patients to urban brownfield EMS park 2 due to distance considerations;
- Existing EMS facilities 1, 2, 4, 11, 12, and 13 have capacities of 250, 250, 330, 250, 261, and 350, respectively. These facilities can potentially be transformed into isolation centers during major public health emergencies. Transfers from these large-scale facilities will be partial to reduce the burden on any single facility. Otherwise, it will pose a greater threat to urban brownfield EMS parks receiving transferred patients once an internal emergency occurs. Each will transfer patients to two designated urban brownfield parks: EMS facility 1 to urban brownfield EMS parks 2 and 9; EMS facility 2 to urban brownfield EMS parks 8 and 9; EMS facility 4 to urban brownfield EMS parks 8 and 9; EMS facility 11 to urban brownfield EMS parks 1 and 2; EMS facility 12 to urban brownfield EMS parks 3 and 9; and EMS facility 13 to urban brownfield EMS parks 5 and 9.

Previous studies by Jientrakul et al. (2022)^57^, Zhu et al. (2021)^58^, and Chen et al. (2016)^59^ focused on optimizing ambulance allocation to ensure patients reach the nearest EMS facility but did not address whether these facilities could accommodate all transferred patients. In major public health emergencies, most city EMS facilities may exceed their maximum capacity. Therefore, it is crucial to redirect some pre-diagnosed patients to large-scale isolation medical services in outlying EMS facilities. The "consultation and referral" model proposed in this study addresses this gap by considering both transport time and the capacity of each EMS facility, ensuring transferred patients are properly accommodated without needing further transfers.

## Conclusions

This study discusses a specific evaluation method for existing EMS systems using the service radii of existing EMS facilities. This parameter, combined with the characteristics of Huangshi, was applied for analysis and selection of urban brownfields to be transformed into EMS parks. This approach would improve the urban EMS system, and effectively solve the problem of remediating urban brownfields in the course of the urban upgrading of Huangshi. It provides new insights into the urban upgrading of mining cities. This study may also be significant for the construction of EMS systems in other mining cities and help improve EMS system globally. However, this study did not address the differences between the EMS systems of mining cities and conventional cities; it used only data from the COVID-19 outbreak and did not include the data from other major public health emergency systems. This model needs further studies and improvement to be applicable to other systems.

### Supplementary Information

Supplementary Tables.

## Data Availability

The datasets used and/or analysed during the current study available from the corresponding author on reasonable request.
