# Supplementary material for: Improving urban emergency medical service systems through brownfield transformation in Huangshi, China
Source: Sci Rep. 2024 Jun 28;14:14946. doi: 10.1038/s41598-024-66080-3 (PMC11213939; doi:10.1038/s41598-024-66080-3)
Supplement: Supplementary file 1 — Supplementary Tables. [file 41598_2024_66080_MOESM1_ESM.docx]

**Supplementary Information**

**Table S1** Area, population, population density, and cumulative number of confirmed cases of each district of Huangshi

**Table S2** Capacities of existing EMS facilities in Huangshi

**Table S3** Coordinates and areas of urban brownfields

**Table S4** Capacities and service radii of existing EMS facilities

**Table S5** Capacities and service radii urban brownfield EMS parks

**Table S1** Area, population, population density, and cumulative number of confirmed cases of each district of Huangshi

| No. | District | Area (km^2^) | Population (10 thousand) | Population density (person/km^2^) | Cumulative number of confirmed cases (person) |
| --- | --- | --- | --- | --- | --- |
| 1 | Xialu | 69.0 | 21.52 | 3118.84 | 134 |
| 2 | Xisaishan | 100.0 | 19.72 | 1972.00 | 146 |
| 3 | Haungshigang | 30.0 | 24.16 | 8053.33 | 203 |
| 4 | Tieshan | 28.0 | 4.19 | 1496.43 | 92 |
| 5 | Daye | 1566.0 | 87.12 | 556.32 | 241 |
| 6 | Yangxin | 2783.0 | 90.19 | 324.07 | 161 |

**Table S2** Capacities of existing EMS facilities in Huangshi

| No. | Existing EMS facilities | Temporary observation patient | Mild patient | Severe patient |
| --- | --- | --- | --- | --- |
| 1 | Huangshi youse hospital | 0 | 250 | 0 |
| 2 | Huangshi kuangwuju hospital | 0 | 250 | 0 |
| 3 | Huangshi central hospital（Puai ward） | 0 | 0 | 24 |
| 4 | Huansghi hospital of traditional Chinese medicine | 0 | 311 | 19 |
| 5 | Huangshi maternity & child health hospital | 10 | 0 | 0 |
| 6 | The second hospital of Huangshi | 9 | 0 | 0 |
| 7 | The fourth hospital of Huangshi | 11 | 0 | 0 |
| 8 | The fifth hospital of Huangshi | 12 | 0 | 0 |
| 9 | Huangshi puren hospital | 9 | 0 | 0 |
| 10 | Huangshi huaxin hospital | 9 | 0 | 0 |
| 11 | Daye tiekuang hospital | 0 | 235 | 15 |
| 12 | Daye hospital of traditional Chinese medicine | 0 | 245 | 16 |
| 13 | Yangxin people’s hospital | 0 | 330 | 20 |

**Table S3** Coordinates and areas of urban brownfields

| No. | Longitude | Latitude | Area (km^2^) |
| --- | --- | --- | --- |
| 1 | 114.709042 °E | 30.269611 °N | 0.14 |
| 2 | 114.742802 °E | 30.133001 °N | 0.04 |
| 3 | 114.723984 °E | 29.980165 °N | 0.06 |
| 4 | 114.784075 °E | 29.982672 °N | 0.07 |
| 5 | 114.900528 °E | 29.807171 °N | 0.14 |
| 6 | 115.048647 °E | 29.679869 °N | 0.19 |
| 7 | 115.135658 °E | 29.750375 °N | 0.04 |
| 8 | 115.270407 °E | 30.096111 °N | 0.15 |
| 9 | 115.207632 °E | 30.154357 °N | 0.20 |

**Table S4** Capacities and service radii of existing EMS facilities

| No. of existing EMS facilities | Capacities (person) | Service radii (m) |
| --- | --- | --- |
| 1 | 250 | 3994.37 |
| 2 | 250 | 4609.72 |
| 3 | 24 | 1428.27 |
| 4 | 330 | 2492.84 |
| 5 | 10 | 798.87 |
| 6 | 9 | 411.68 |
| 7 | 11 | 664.61 |
| 8 | 12 | 875.12 |
| 9 | 9 | 411.68 |
| 10 | 9 | 411.68 |
| 11 | 250 | 8718.22 |
| 12 | 261 | 14609.79 |
| 13 | 350 | 29323.77 |

**Table S5** Capacities and service radii urban brownfield EMS parks

| No. of urban mining brownfield EMS parks | Capacities (person) | Service radii (m) |
| --- | --- | --- |
| 1 | 281 | 15153.46 |
| 2 | 80 | 8099.86 |
| 3 | 120 | 9920.27 |
| 4 | 240 | 10715.11 |
| 5 | 281 | 26264.77 |
| 6 | 381 | 30597.53 |
| 7 | 80 | 14039.11 |
| 8 | 301 | 5056.78 |
| 9 | 401 | 5839.07 |
